# Multiple Irrelevant Duration Information Affects the Perception of Relevant Duration Information: Interference With Selective Processing of Duration

**DOI:** 10.1177/2041669520973223

**Published:** 2020-11-26

**Authors:** Hitomi Kawahara, Yuko Yotsumoto

**Affiliations:** Department of Integrated Sciences, The University of Tokyo, Tokyo, Japan; Department of Life Sciences, The University of Tokyo, Tokyo, Japan

**Keywords:** time perception, duration perception, interval timing, attention, distractors

## Abstract

In the human visual environment, the ability to perceive only relevant duration is important for various activities. However, a relatively small number of studies have investigated how humans process multiple durations, in comparison with the processing of one or two durations. We investigated the effects of multiple irrelevant durations on the perception of relevant duration. In four behavioral experiments, the participants were instructed to pay attention to a target stimulus while ignoring the distractors; then, they reproduced the target duration. We manipulated three aspects of the distractors: number, duration range, and cortical distance to the target. The results showed that the presence of multiple irrelevant durations interfered with the processing of relevant duration in terms of the mean perceived duration and the variability of the perceived duration. The interference was directional; that is, longer (shorter) irrelevant durations made the reproduced durations longer (shorter). Moreover, the interference was not likely to depend on the cortical distance between the target and the distractors, suggesting an involvement of relatively higher cortical areas. These results demonstrate that multiple irrelevant duration information affects the temporal processing of relevant duration information and suggest that multiple independent clocks assigned to each of the durations may not exist.

Processing temporal information is an essential ability in human activities such as speech, motor control, or playing music. Many studies have investigated how humans or other animals process temporal information, and how subjective time is constructed in our brain. Accumulated evidence on human time perception indicates that subjective time is influenced by various nontemporal factors, such as stimulus intensity (Goldstone et al., 1978; Gomez & Robertson, 1979), attention (Brown & Stubbs, 1992; Ono et al., 2007), and memory (Bi et al., 2014; Pan & Luo, 2012). Based on such observations, researchers have proposed models on time perception. The pacemaker-accumulator framework is one of the most frequently adopted models by time-perception researchers. This model assumes that a pacemaker in our system emits constant pulses to an accumulator, and subjective time is thus determined by the number of pulses collected in the accumulator (Creelman, 1962; Treisman, 1963).

While the human visual environment contains a variety of temporal information, most previous studies have only examined the perception of duration of one or two simple stimuli (Allan & Gibbon, 1991; Droit-Volet, 2008; Goldstone et al., 1978), and how humans process more than a few stimuli at the same time is still not fully understood. Based on the pacemaker-accumulator framework, van Rijn and Taatgen (2008) proposed three possible systems underlying the processing of multiple temporal information. Such models consider how many pacemakers and accumulators exist and process multiple temporal information (Crystal, 2003; Rousseau & Rousseau, 1996; van Rijn & Taatgen, 2008). Empirical data implied that it can be hard or almost impossible to process multiple durations independently and simultaneously and suggested that multiple independent pacemakers and accumulators are not likely to exist (Ayhan et al., 2012; Brown & Stubbs, 1992; Brown & West, 1990; Bryce et al., 2015). Brown and West (1990) examined the ability to memorize multiple intervals and showed that the accuracy of temporal processing decreased as the number of simultaneously presented stimuli increased. More recently, Ayhan et al. (2012) reported that the discrimination thresholds of the target duration increased when eight distractors surrounded the target stimulus. These studies demonstrated that distractor stimuli surrounding a target stimulus can interfere with the temporal processing of the target. Morgan et al. (2008) also reported a similar interference effect. However, the detailed characteristics of the interference have not yet been examined.

Attention to the stimulus modulates its temporal processing. Previous studies have reported that an attended stimulus was perceived to be longer than unattended ones (Enns et al., 1999; Mattes & Ulrich, 1998; Yeshurun & Marom, 2008), which is mainly explained by the attentional facilitation of temporal processing of the attended stimulus (Matthews & Meck, 2016). On the other hand, temporal processing can become less accurate if other factors reduce the amount of attentional resources allocated to such temporal processing. For example, concurrently conducting a nontemporal and a temporal task should decrease the amount of attention directed to the temporal task. Many studies have demonstrated that perceived durations become shorter and more variable in such dual-task conditions (Block et al., 2010; Brown, 1985; Brown & Stubbs, 1992; Hallez & Droit-Volet, 2019). Therefore, attention plays an essential role in temporal processing. These previous studies examined the effects of attention while participants observed only up to two temporal stimuli. However, the effects of attention on duration perception should be examined in a more realistic situation, where multiple sources of information appears with various timings.

The aim of the present study was to systematically explore whether, and which aspects of, multiple irrelevant duration information may affect the processing of the duration of a single relevant stimulus. We conducted four behavioral experiments to examine the effects of multiple visual distractors (irrelevant duration) on the perception of duration of a target stimulus (relevant duration) while manipulating several aspects of the distractors (number, duration range, and spatial location). In the experiments, participants performed a duration reproduction task, in which they had to reproduce the duration of the target stimulus (Mioni et al., 2014). In a trial, a visual target stimulus and multiple visual distractors were presented with various onsets, offsets, and durations (for a schematic experimental procedure, see Figure 1A and B). Each stimulus duration temporally overlapped with the durations of the other stimuli (Figure 1C). Participants were presented with both the target and distractors while instructed to pay attention only to the target while ignoring the distractors. After all the stimuli disappeared, participants reproduced the target duration by pressing a key. We focused on two aspects of temporal perception: mean perceived duration (normalized reproduced duration) and variability of the perceived duration measured as the coefficient of variation (CV). Mean perceived duration indicates how well, on average, the processing of duration in a human timing system can approximate the duration to be estimated, while the variability of perceived duration reveals how stable the processing is (Gamache & Grondin, 2010; Grondin & McAuley, 2009; Mioni et al., 2016). An accurate perception of duration should be close to the actual duration in terms of mean perceived duration and stable in terms of variability.

**Figure 1.**
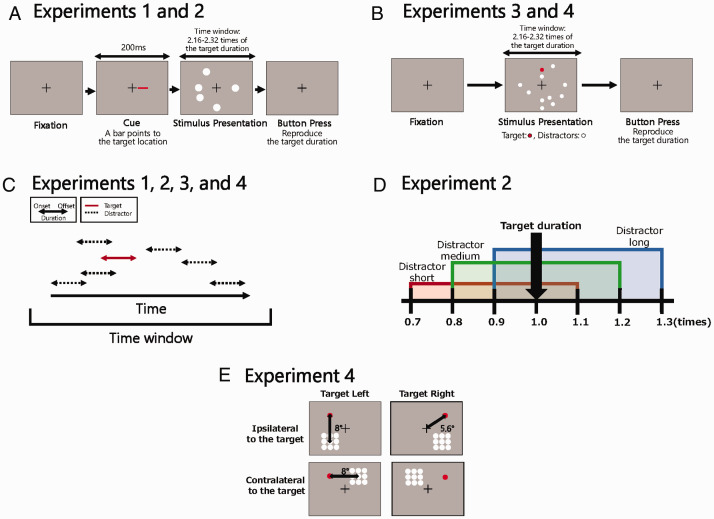
Schematic Illustrations of the Task. A: Experimental procedure of Experiments 1 and 2. B: Experimental procedure of Experiments 3 and 4. C: Schematic timetable of the stimulus presentation. In this illustration, a solid arrow indicates the target duration, and the other dashed arrows indicate distractors durations. D: Duration ranges of distractors. E: Stimulus configuration of Experiment 4.

In Experiment 1, we examined whether the mere presence of distractors itself affected the perception of the target duration by manipulating the number of distractors surrounding the target stimulus. In Experiment 2, we manipulated the duration range of the distractors (Figure 1D) and tested whether the number and durations of the distractors would affect the perception of the target duration. In Experiment 3, we increased the number of distractors relative to Experiment 1 to measure the limit of the interference observed in Experiment 1. Finally, in Experiment 4, we set the distractors on the same visual field (ipsilateral) or different visual field (contralateral) relative to the target stimulus (Figure 1E) to find out whether the distractors cortically close to the target showed more robust interfering effects on the temporal processing of the target duration, compared with the distractors cortically remote to the target.

## Experiment 1

In Experiment 1, we examined whether the mere presence of multiple distractors affected the perception of duration of a target stimulus by manipulating the number of distractors (0, 3, 6, 9, or 11). Participants were presented with both the target and distractors and instructed to pay attention to the target while ignoring the distractors. After all the stimuli disappeared, participants reproduced the duration of the target stimulus (i.e., target duration: 450, 600, or 750 ms) using a continuous button press.

### Methods

#### Participants

Data were collected from 16 participants (14 males, age [years] mean [*M*] = 21.9, standard deviation [*SD*] = 2.3) in Experiment 1. For each experiment (Experiments 1 to 4), 14 to 16 participants were recruited. G*Power analyses (Faul et al., 2009) suggested that a total sample size of 14, 7, or 8 was sufficient to detect the effect of *f* = 0.25 (medium effect size) with 80% power in a 3 × 5, 3 × 5 × 2, or 3 × 4 × 2 within-subjects analysis of variance (ANOVA; alpha = .05, nonsphericity correction = 1), respectively. We assumed the medium effect size, which is more conservative compared with the large effect size reported in a previous study with a similar experimental design (Ayhan et al., 2012; $\eta_{\text{p}}^{2}$ = 0.80, *f* = 2.01). All participants in this study had normal or corrected-to-normal vision and provided written informed consent to participate in the experiment in accordance with the Declaration of Helsinki. The protocol was approved by the institutional review boards of the University of Tokyo, and all experiments were carried out in accordance with the guidelines set by the Ethics Committee of the University of Tokyo.

#### Apparatus

The experiment was conducted in a darkened room. Participants sat in front of a Sony CPD-E230 15.4-inch cathode ray tube monitor (screen resolution at 1,024 × 768 pixels, 85 Hz refresh rate) at a distance of 57.3 cm, with their heads on a chin rest for head stability. The experiment was conducted with MATLAB 2017b (The MathWorks Inc., Natick, MA, USA) using Psychophysics Toolbox extensions (Kleiner et al., 2007).

#### Stimuli

A white fixation cross was presented in the center of the display against a gray background. The experiment used two kinds of stimuli: target and distractors. In Experiment 1, both the target and distractor were a white Gaussian blob with a radius of 1.5°. A red bar was used to indicate the location of the target, and participants were presented with the target and the distractors, which had completely identical features. The target and distractors randomly appeared at 1 of the 12 locations on an invisible circle. The distance between the center of the stimulus and the center of the display (eccentricity) was 8°.

#### Procedure

Participants performed a temporal reproduction task (Mioni et al., 2014), where they reproduced the duration of the target stimulus (Figure 1A). When a trial started, a white fixation cross appeared at the center of the display. After the location of the target was indicated by a red bar for 200 ms, the single target and multiple distractors (0, 3, 6, 9, or 11 in number) asynchronously appeared on the display within a time window for various durations with different onsets and offsets (for a schematic illustration, see Figure 1C). The time window was determined from a uniform distribution between 2.16 and 2.32 times of the target duration. We instructed the participants to pay attention only to the target stimulus while ignoring the distractors, keeping their gaze on the fixation cross. After all the stimuli disappeared, participants reproduced the duration of the target stimulus (450, 600, or 750 ms) by continuously pressing a space bar on a keyboard. Participants were instructed not to count (Clément & Droit-Volet, 2006; Rattat & Droit-Volet, 2012). In Experiment 1, a 500-ms brief break was inserted before participants reproduced the target duration, which was eliminated after Experiment 2 for task simplicity. The trial ended when the space key was released. The intertrial interval was set to 250 to 500 ms.

The target duration and number of distractors determined the condition for each trial. Although the target durations used in analyses were 450, 600, and 750 ms, there were catch trials with other target durations to prevent participants from learning the specific target durations; 25% of the trials were catch trials. The catch trials were included in all of the experiments in the present study. The target duration in the catch trials was sampled from the uniform distributions of 400–440, 460–590, 610–740, or 760–800 ms. Each condition was displayed 40 times in Experiment 1. Three durations of the target stimulus (450, 600, 750 ms) and five numbers of distractors (0, 3, 6, 9, or 11) resulted in 600 trials. With 200 catch trials, the total number of trials was 800. Experiment 1 involved one session, which included four blocks. Participants experienced all the conditions (within-subjects design).

There were two independent variables in Experiment 1: target duration (450, 600, or 750 ms) and number of distractors (0, 3, 6, 9, or 11). The duration of each distractor was extracted randomly from the uniform distribution that ranged from 0.8 to 1.2 times the target duration. For example, if the target duration was 450 ms, the distractor durations were extracted from 360 ms to 540 ms (see Figure 1D). The onsets and offsets of the target and distractors were randomly determined.

#### Data Analysis

We used the normalized reproduced duration and CV as dependent variables. The normalized reproduced duration measured the average perceived duration, while CV indicated the variability of the perceived duration. These values were calculated for each condition for each participant. The normalized reproduced duration was calculated by dividing the mean reproduced duration by the target duration. If the normalized reproduced duration exceeds 1, it indicates an overestimation of the target duration; if it is lower than 1, it indicates an underestimation of the target duration. CV was calculated by dividing the SD of the reproduced duration by the mean reproduced duration (Lewis & Miall, 2009). A larger CV reflects unstable or noisier temporal processing.

For statistical analyses, we conducted a 3 × 5 repeated-measures analysis of variance for each dependent variable (i.e., normalized reproduced duration and CV), with target duration (450, 600, or 750 ms) and number of distractors (0, 3, 6, 9, or 11) as within-subjects factors, using R (R Development Core Team, 2020). Post hoc tests with a modified sequentially rejective Bonferroni correction (Shaffer, 1986) were performed to examine the ANOVA contrasts. The effect size was estimated using partial eta squared ($\eta_{\text{p}}^{2}$).

Responses more than two interquantile ranges below the first quantile, or two interquantile ranges above the third quantile were regarded as outliers; accordingly, 2.7% of the total responses were excluded from further analyses in Experiment 1.

### Results

#### Normalized Reproduced Duration

**Figure 2.**
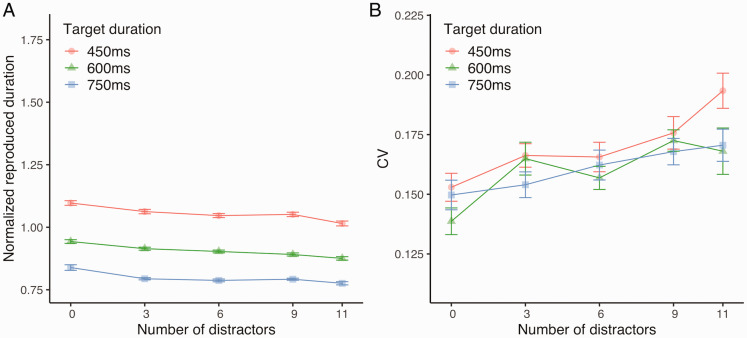
Effects of Number of Distractors on the Temporal Processing of the Target Stimulus (Experiment 1). In all figures, each target duration is allocated different shapes (circle: 450 ms, triangle: 600 ms, square: 750 ms). The *x* axis indicates the number of distractors presented around the target stimulus. The error bars are within-subjects standard errors (Loftus & Masson, 1994; Masson, 2004). A: Normalized reproduced duration. Larger values indicate longer reproductions of the target duration. B: CV. Larger values indicate larger variability of the duration reproductions. CV = coefficient of variation.

Figure 2A shows the normalized reproduced duration; the error bars are within-subjects standard errors (Loftus & Masson, 1994; Masson, 2004). The 3 × 5 repeated-measures ANOVA conducted on the normalized reproduced duration revealed a significant main effect of the number of distractors, *F*(4, 60) = 17.19, *p* < .001, $\eta_{\text{p}}^{2}$ = 0.53, and a significant main effect of the target duration, *F*(2, 30) = 81.51, *p* < .001, $\eta_{\text{p}}^{2}$ = 0.84. No significant interactions between the number of distractors and target duration were found, *F*(8, 120) = 1.01, *p* = .42, $\eta_{\text{p}}^{2}$ = 0.06. Post hoc pairwise comparisons revealed that the normalized reproduced duration was significantly longer when the number of distractors was 0 than when the number of distractors was 3, 6, 9, or 11 and that the normalized reproduced duration was shorter when the number was 11 than when the number of distractors was 3 or 9 (all *p*s < .05), suggesting that the normalized reproduced duration decreased as the number of distractors increased. With respect to the main effect of the target duration, post hoc pairwise comparisons indicated that the normalized reproduced duration was largest when the target duration was 450 ms, followed by 600 and 750 ms (all *p*s < .001), suggesting that participants reproduced the target duration of 450 ms as longer and the duration of 750 ms as shorter than the actual durations. This response tendency is already known as the “central-tendency effect,” whereby participants tend to over-reproduce “shorter” durations and under-reproduce “longer” durations when they have experienced a range of durations (Jazayeri & Shadlen, 2010).

#### CV

Figure 2B shows the CV. The 3 × 5 repeated-measures ANOVA conducted on CV revealed a significant main effect of the number of distractors, *F*(4, 60) = 6.11, *p* < .001, $\eta_{\text{p}}^{2}$ = 0.30. Neither the main effect of the target duration, *F*(2, 30) = 1.83, *p* = .18, $\eta_{\text{p}}^{2}$ = 0.11, nor the interaction between the number of distractors and target duration, *F*(8, 120) = 0.94, *p* = .49, $\eta_{\text{p}}^{2}$ = 0.06, was significant, suggesting that CV did not depend on the duration of the target stimulus. Post hoc pairwise comparisons revealed that CV was significantly smaller when the number of distractors was 0 than when the number of distractors was 9 or 11 (all *ps* < .05), suggesting that CV increased as the number of distractors increased.

In sum, Experiment 1 revealed that as the number of distractors increased, the temporal processing of the target stimulus became noisier, and the reproduced duration was slightly shortened. These findings indicate the interference of the multiple distractors in the selective temporal processing of the target.

## Experiment 2

Experiment 1 showed that the presence of irrelevant distractors impaired the processing of relevant duration information. In Experiment 1, each distractor lasted for 0.8–1.2 times the target duration, and the average duration of the distractors was almost the same as that of the target. Therefore, the participants could reproduce the target duration by averaging all the stimuli including the distractors. In Experiment 2, we manipulated the duration range of the distractors and further evaluated the effect of the distractors on the mean reproduced duration. In Experiment 2, we set the duration range of distractors to be either 0.7–1.1 or 0.9–1.3 times the target duration. In other words, distractors lasted, on average, shorter or longer than the target. If the length of the duration of distractors has no effect on the length of the reproduced duration of the target, there should be no difference whether the duration range of the distractors was relatively longer or shorter than the target duration.

### Methods

#### Participants

Data were collected from 14 participants (11 males, age [years] *M* = 21.5, *SD* = 0.96) in Experiment 2. None of them had participated in the previous experiment.

#### Apparatus

The same materials as in Experiment 1 were used in Experiment 2.

#### Stimuli

Stimuli were identical to those used in Experiment 1.

#### Procedure

The task procedure was almost the same as in Experiment 1, except that the 500-ms blank before the key press was eliminated for task simplicity in Experiment 2. The independent variables in Experiment 1 (target duration and number of distractors) were also manipulated in Experiment 2, but the main independent variable was the duration range of distractors. There were two distractor duration ranges: one distributed from 0.7 to 0.1 times the target duration, and the other distributed from 0.9 to 1.3 times the target, meaning that distractors lasted shorter or longer on average than the target stimulus did (Figure 1D).

The target duration, number of distractors, and duration range determined the condition for each trial, and each condition was displayed 40 times in Experiment 2, resulting in a total of 1,600 trials. Experiment 2 consisted of two sessions, each comprising 20 blocks. Participants experienced all the conditions (within-subjects design).

#### Data Analysis

Normalized reproduced duration and CV were calculated in the same way as in Experiment 1. For statistical analyses, normalized reproduced duration and CV were analyzed in 3 × 4 × 2 repeated-measures ANOVAs. Only conditions that presented distractors more than 0 were included in ANOVAs because there should not be differences between 0 distractor conditions depending on the duration range of distractors. Alternatively, normalized reproduced duration and CV in 0 distractor conditions were compared with those of other conditions, in terms of the number of distractors and the target duration, using post hoc multiple comparison tests. Post hoc tests were performed in the same way as in Experiment 1. Responses more than two interquantile ranges from the first quantile or above two interquantile ranges from the third quantile were regarded as outliers; thus, 1.3% of the total responses were excluded from further analyses in Experiment 2.

### Results

#### Normalized Reproduced Duration

**Figure 3.**
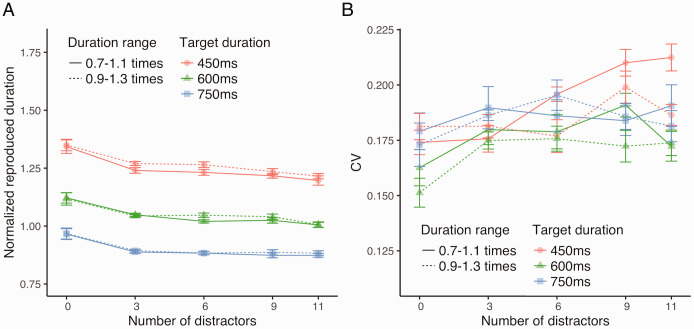
Effects of the Duration Range of Distractors (Experiment 2). The dashed lines indicate the condition where distractors lasted longer on average, while the solid lines indicate the condition where the distractors had a shorter duration on average, compared with the target duration. A: Normalized reproduced duration. B: CV. CV = coefficient of variation.

Figure 3A shows the normalized reproduced duration. The 3 × 4 × 2 repeated-measures ANOVA conducted on the normalized reproduced duration revealed a significant main effect of the number of distractors, *F*(3, 39) = 8.85, *p* = .001, $\eta_{\text{p}}^{2}$ = 0.41, the target duration, *F*(2, 26) = 73.29, *p* < .001, $\eta_{\text{p}}^{2}$ = 0.85, the duration range of distractors, *F*(1, 13) = 6.79, *p* = .02, $\eta_{\text{p}}^{2}$ = 0.34, and a significant interaction between the number and duration range of distractors, *F*(3, 39) = 13.67, *p* < .001, $\eta_{\text{p}}^{2}$ = 0.51. With respect to the significant main effect of the target duration, post hoc pairwise comparisons indicated that normalized reproduced duration was largest when the target duration was 450 ms, followed by 600 and 750 ms (all *p*s < .001). Following the significant interaction between the number and duration range of distractors, post hoc tests revealed a simple main effect of the number of distractors for conditions with short distractor duration, *F*(3, 39) = 12.03, *p* < .001, $\eta_{\text{p}}^{2}$ = 0.48. Then, pairwise comparisons following the simple main effect of the number of distractors showed that the normalized reproduced duration was longer when the number of distractors was 3 compared with when the number was 6, 9, or 11 and that the normalized reproduced duration was longer when the number was 6 compared with when the number was 9 or 11 (all *p*s < .05). This further suggests that the normalized reproduced duration decreased as the number of distractors increased, if the duration of distractors was 0.7 to 1.1 times as long as the target duration. Analyses of simple main effects also revealed significant simple main effects of the duration range of distractors when the number of distractors was 3, *F*(1, 13) = 12.90, *p* = .003, $\eta_{\text{p}}^{2}$ = 0.50, and 6, *F*(1, 13) = 14.56, *p* = .002, $\eta_{\text{p}}^{2}$ = 0.53, suggesting that the normalized reproduced duration was shorter when the duration of distractors was 0.7 to 1.1 times as long as the target duration than when the duration was 0.9 to 1.3 times as long as the target duration. When the number of distractors was 0, the normalized reproduced duration was significantly larger compared with when the number was 3 or 11 (both *p*s < .05).

#### CV

Figure 3B shows the CV. The 3 × 4 × 2 repeated-measures ANOVA conducted on CV revealed a significant main effect of the number of distractors, *F*(3, 39) = 4.22, *p* = .01, $\eta_{\text{p}}^{2}$ = 0.24, and the significant interaction between the number and duration range of distractors, *F*(3, 39) = 3.49, *p* = .02, $\eta_{\text{p}}^{2}$ = 0.21. Following the significant interaction, analyses of simple main effects revealed a simple main effect of the number of distractors for conditions with short distractor duration, *F*(3, 39) = 7.64, *p* < .001, $\eta_{\text{p}}^{2}$ = 0.37. Multiple comparisons with respect to the significant simple main effect of number of distractors indicated that CV was smaller when the number of distractors was 3 than when the number was 9 or 11 and that CV with 6 distractors was smaller than that with 9 distractors (all *p*s < .05), suggesting that CV increased as the number of distractors increased. CV when the number of distractors was 0 was not significantly larger or smaller than that when the number was 3, 6, or 9 (all *p*s > .05).

Experiment 2 revealed that the reproduced duration of the target stimulus was affected by the surrounding durations such that the reproduced duration was pulled toward the average distractor durations. The results suggest that the durations of the multiple irrelevant stimuli, as well as their mere presence, influence the duration perception of the relevant information.

## Experiment 3

Experiments 1 and 2 examined the number of distractors from 0 to 11 and found that the number of distractors affected the duration perception of the target in terms of both normalized reproduced duration and CV. Yet, it is possible that there may be a point where an increased number of distractors would not affect the participants’ time reproduction anymore, even if the number of distractors increases further than that in Experiments 1 and 2. Therefore, in Experiment 3, we increased the number of distractors up to 79.

### Methods

#### Participants

Data were collected from 14 participants (11 males, age [years] *M* = 20.6, *SD* =1.4) in Experiment 3; 2 of the 14 participants had taken part in Experiment 1.

#### Apparatus

The same materials as in Experiments 1 and 2 were used in Experiment 3.

#### Stimuli

A white fixation cross was presented in the center of the display against a gray background. In Experiments 3 and 4, the target and distractor were discriminated by using a red Gaussian blob with a radius of 0.5° (Experiment 3) or 1° (Experiment 4) as the target.

The stimuli appeared at 1 of 80 locations embedded in four invisible circles. The smallest circle contained 8, the second 16, the third 24, and the forth 32 locations, and the distances between the center of the stimulus and the center of the display were 1.6°, 3.2°, 4.8°, or 6.4° on each invisible circle, respectively.

#### Procedure

The task was almost identical to that in Experiments 1 and 2, although the color of the target stimulus was red in Experiment 3 (Figure 1B).

When a trial started, a white fixation cross appeared on the center of the display. Next, the target and distractors asynchronously appeared on the display within a time window for various durations with various onsets and offsets. We instructed the participants to pay attention only to the red target stimulus while ignoring the white distractors. After all the stimuli disappeared, participants reproduced the duration of the target stimulus by continuously pressing the space bar on a keyboard. Participants were instructed not to count.

The same independent variables as in Experiment 1 (target duration and number of distractors) were manipulated in Experiment 3. However, the number of distractors was increased (0, 10, 20, 40, or 79) in Experiment 3, compared with Experiment 1 (0, 3, 6, 9, or 11).

The target duration and number of distractors determined the condition for each trial. Each condition was displayed 40 times in Experiment 3, resulting in a total of 800 trials. Experiment 3 consisted of two sessions, each including 10 blocks. Participants experienced all the conditions (within-subjects design).

#### Data Analysis

We calculated the normalized reproduced duration and CV. For statistical analyses, we conducted a 3 × 5 repeated-measures ANOVA for each dependent variable (normalized reproduced duration and CV), with target duration (450, 600, or 750 ms) and number of distractors (0, 10, 20, 40, or 79) as within-subject factors. Post hoc tests were performed in the same way as in Experiments 1 and 2. Responses more than two interquantile ranges from the first quantile or above two interquantile ranges from the third quantile were regarded as outliers; thus, 2.4% of the total responses were excluded from further analyses in Experiment 3.

### Results

#### Normalized Reproduced Duration

**Figure 4.**
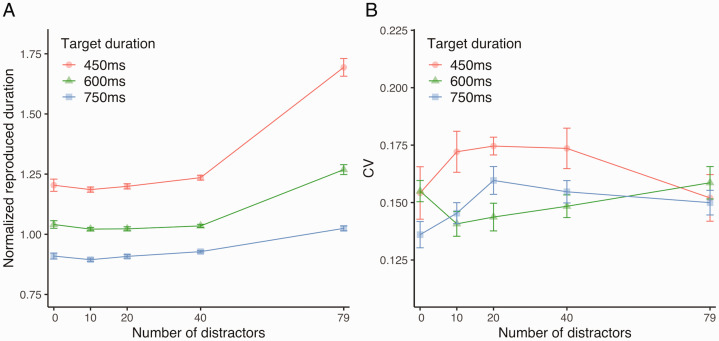
Effects of the Number of Distractors (Experiment 3). A: Normalized reproduced duration. B: CV. CV = coefficient of variation.

Figure 4A shows the normalized reproduced duration. The 3 × 5 repeated-measures ANOVA conducted on the normalized reproduced duration revealed a significant main effect of the number of distractors, *F*(4, 52) = 72.38, *p* < .001, $\eta_{\text{p}}^{2}$ = 0.85, a significant main effect of the target duration, *F*(2, 26) = 265.66, *p* < .001, $\eta_{\text{p}}^{2}$ = 0.95, and a significant interaction between the number of distractors and target duration, *F*(8, 104) = 62.05, *p* < .001, $\eta_{\text{p}}^{2}$ = 0.83. Following the significant interaction between the number of distractors and target duration, post hoc tests revealed a simple main effect of the number of distractors for each target duration (all *ps* < .001) and a simple main effect of the target duration for each number of distractors (all *ps* < .001). Post hoc pairwise comparisons following the simple main effect of the target duration revealed that the normalized reproduced duration was the largest when the target duration was 450 ms, followed by 600 ms and 750 ms (all *ps* < .001). Regarding the simple main effect of the number of distractors, for each target duration, the normalized reproduced duration with 79 distractors was significantly longer than that with 0, 10, 20, or 40 distractors (all *ps* < .05).

#### CV

Figure 4B shows the CV. The 3 × 5 repeated-measures ANOVA conducted on CV revealed a significant main effect of target duration, *F*(2, 26) = 3.49, *p* = .05, $\eta_{\text{p}}^{2}$ = 0.21, and a nonsignificant main effect of number of distractors, *F*(4, 52) = 0.6781, *p* = .06, $\eta_{\text{p}}^{2}$ = 0.05, with a significant interaction of target duration and number of distractors, *F*(8, 104) = 2.37, *p* = .02, $\eta_{\text{p}}^{2}$ = 0.15. Following the significant interaction, analysis of simple effects revealed a significant simple main effect of target duration on CV when the number of distractors was 10, *F*(2, 26) = 3.45, *p* = .05, $\eta_{\text{p}}^{2}$ = 0.21, or 40, *F*(2, 26) = 4.50, *p* = .02, $\eta_{\text{p}}^{2}$ = 0.26. However, overall, there are no consistent patterns in the relationship between the number of distractors and CVs across different target durations.

## Experiment 4

In some cases, interferences across multiple visual items show location-dependent characteristics. Neurons in early visual cortices have small receptive fields and retinotopic representations (Zeki, 1978). When two stimuli are presented within one (left or right) visual field, the two stimuli are first processed within one (right or left) hemisphere. When two stimuli are presented in different visual fields, one is processed in the left hemisphere and the other in the right hemisphere. Therefore, the cortical representations of the two stimuli in the early visual cortex would be relatively closer when they are presented within one visual field and relatively farther when they are presented in different visual fields. If the amount of interference is larger when the two stimuli are presented within one visual field than when they are presented in different visual fields, such interference may result from processing in the early visual cortical areas (Liu et al., 2009; Pillow & Rubin, 2002). Therefore, by manipulating the locations of the stimuli, we would be able to infer the neural sites underlying the interference.

In the domain of time perception, several studies used the same logic to localize the neural sites of temporal processing. Maarseveen et al. (2017) showed that the effects of the adaptation to the duration occurred irrespective of the cortical distance between the adapter and the test stimulus, with the conclusion that duration information is encoded later on in the visual processing in the case of duration adaptation. On the other hand, Okajima and Yotsumoto (2016) showed that a flickering distractor presented ipsilateral (i.e., within the same visual field) to the target prolonged the perceived duration of the target stimulus more than the contralateral (i.e., across the visual fields) distractor did. In Experiment 4, we manipulated the cortical distance of the target and distractors to examine the neural sites of the interference of the distractors with the target processing. If ipsilateral distractors have larger effects on the duration perception of the target, it is possible that processing in the early cortical areas is involved in the interference of distractors.

### Methods

#### Participants

Data were collected from 16 participants (9 males, age [years] *M* = 20.3, *SD* = 0.75) in Experiment 4. None of them had participated in the previous experiments.

#### Apparatus

The same materials as in Experiments 1, 2, and 3 were used in Experiment 4. Participants sat in front of a cathode ray tube monitor at a distance of 100 cm.

#### Stimuli

In Experiment 4, the same stimuli as in Experiment 3 were used, except that the radius of the stimuli was 1° in Experiment 4. Figure 1E shows the stimulus configurations. The target stimulus was located either at 4° right or left of, and 4° above, the fixation cross, resulting in 5.6° eccentricity. The distractors were displayed either ipsilateral or contralateral to the target in the visual hemifield. The minimum distance between the center of the target and distractor was 6° in both the contralateral and ipsilateral conditions. The distance between the centers of the distractors was 2°. The combination of distractor locations was determined so that the average distance between the center of the distractors and the target was almost identical among the conditions (approximately 8°) to exclude confounding between physical distance and cortical distance.

#### Procedure

The task was identical to that in Experiment 3 (see Figure 1B). The independent variables in Experiment 4 were distance between the target and distractors (ipsilateral or contralateral to the target stimulus), with the target duration being either 450, 600, or 750 ms, and the number of distractors being either 0, 3, 6, or 9. These independent variables determined the condition for each trial. Each condition was displayed 36 times in Experiment 4, resulting in a total of 1,152 trials. Experiment 4 involved two sessions, each of which included nine blocks. Participants experienced all the conditions (within-subjects design).

#### Data Analysis

We calculated the normalized reproduced duration and CV. For statistical analyses, normalized reproduced duration and CV were analyzed in 3 × 3 × 2 repeated-measures ANOVAs. Only conditions that presented distractors more than 0 were included in ANOVAs, because there should not be differences between 0 distractor conditions depending on the distractor locations. Alternatively, normalized reproduced duration and CV in 0 distractor conditions were compared with those of other conditions, in terms of the number of distractors and the target duration, using post hoc multiple comparison tests. Post hoc tests were also performed in the same way as in Experiments 1, 2 and 3. Data from 1 out of 16 participants with outlying values (±3 SD from the CV across participants) were excluded. Responses more than two interquantile ranges from the first quantile or above two interquantile ranges from the third quantile were regarded as outliers; thus, 1.4% of the total responses were excluded from further analyses in Experiment 4.

### Results

#### Normalized Reproduced Duration

**Figure 5.**
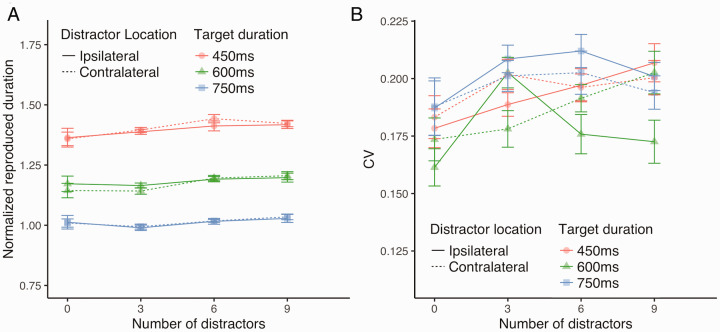
Effects of the Cortical Distance Between the Target and Distractors (Experiment 4). The dashed lines indicate the condition where distractors were placed on the same visual field as the target (“ipsilateral”), and the solid lines refer to the condition where the distractors were located on a different visual field from that of the target (“contralateral”). A: Normalized reproduced duration. B: CV. CV = coefficient of variation.

Figure 5A shows the normalized reproduced duration. The 3 × 3 × 2 repeated-measures ANOVA conducted on the normalized reproduced duration revealed a significant main effect of the target duration, *F*(2, 28) = 48.28, *p* < .001, $\eta_{\text{p}}^{2}$ = 0.78, and the number of distractors, *F*(2, 28) = 3.40, *p* = .05, $\eta_{\text{p}}^{2}$ = 0.20. The main effect of cortical distance between the target and distractors did not reach significance, *F*(1, 14) = 4.27, *p* = .06, $\eta_{\text{p}}^{2}$ = 0.23. Following the significant main effect of the target duration, post hoc pairwise comparisons revealed that the normalized reproduced duration was largest when the target duration was 450 ms, followed by 600 and 750 ms (all *p*s < .001). Pairwise comparisons showed nonsignificant differences among conditions with 3, 6, or 9 distractors (all *p*s > .05). The normalized reproduced duration with 0 distractors was not significantly larger or smaller than those with 3, 6, or 9 distractors (all *p*s > .05).

#### CV

Figure 5B shows the CV. The 3 × 3 × 2 repeated-measures ANOVA conducted on CV did not show a significant main effect of the number of distractors, *F*(2, 28) = 0.54, *p* = .59, $\eta_{\text{p}}^{2}$ = 0.04. Neither the main effect of target duration, *F*(2, 28) = 0.79, *p* = .46, $\eta_{\text{p}}^{2}$ = 0.05, nor cortical distance between the target and distractors, *F*(1, 14) = 2.78, *p* = .12, $\eta_{\text{p}}^{2}$ = 0.17, reached significance. CV with 0 distractors was not significantly larger or smaller than those with 3, 6, or 9 distractors (all *p*s > .05).

To summarize, Experiment 4 showed that whether distractors were located ipsilateral or contralateral to the target (cortical distance) affected neither the normalized reproduced duration nor CV.

## General Discussion

The aim of the study was to investigate the effects of multiple irrelevant duration information on the temporal processing of relevant information. We demonstrated that multiple irrelevant duration information impaired the processing of the relevant duration in terms of mean perceived duration (normalized reproduced duration) and variability of perceived duration (CV). Through four experiments, we demonstrated that multiple irrelevant duration information impaired the processing of the relevant duration in terms of mean perceived duration (mean normalized reproduced duration) and variability of perceived duration (CV). First, the presence of three to nine distractors (i.e., multiple irrelevant duration) made the duration reproduction of the target stimulus (i.e., the relevant duration) variable, while it shortened the normalized reproduced duration (Experiment 1). Second, the reproduced duration of the target was pulled toward the durations of the distractors (Experiment 2). Third, the large number of distractors increased the reproduced duration of the target duration (Experiment 3). Fourth, we did not observe effects of the cortical distance between the target and distractors on the interferences from the distractors (Experiment 4).

Previous studies reported that multiple duration information interfered with the processing of a single target duration. Ayhan et al. (2012) found that the mere presence of multiple distractors increased the duration discrimination variability of two stimuli, while the mean perceived duration of the target stimulus remained stable. Our findings in Experiments 1 and 2 (Figures 2 and 3) are consistent with these previous observations, provided that CV reflects the sensitivity of the temporal system (Gamache & Grondin, 2010). By systematically manipulating characteristics of multiple duration information, our results confirmed that the average perceived duration remained relatively intact, while its precision was impaired by the irrelevant distractors.

It is possible that the presence of distractors interfered with the selective attentional processing of the target duration, making the perceived duration more variable and slightly shorter (Experiments 1 and 2, Figures 2 and 3). Previous studies suggested that selective attention plays an essential role in duration perception (Block et al., 2010; Brown & Stubbs, 1992; Mioni et al., 2016). The attentional gate model (Block & Zakay, 1996), which is based on the pacemaker-accumulator framework, posits a positive correlation between the amount of attentional resources and the performance in temporal tasks. According to this model, when participants are required to divide their attention between a temporal task and other tasks, fewer pulses can pass through the attentional gate and be accumulated, which leads to a more variable and shortened time perception.

We can use the framework of the attentional gate model to interpret the results of Experiments 1 and 2: Attention to the target duration was dispersed by the distractors, which hindered the internal pulses from passing through the attentional gate and induced the variable and slightly shortened perceived duration. However, in Experiment 4, the normalized reproduced duration was not affected by the number of distractors. This difference could result from the distinctiveness (salience) of the target relative to the distractors. According to Fecteau and Munoz (2006), we can very easily detect the presence of the target when it has a distinct feature compared with the surrounding distractors. In Experiment 4, only the target was colored in red, while the distractors were all white. It is possible that this distinctiveness of the target enabled participants to easily detect it and thus effectively direct their attention to it. Moreover, the locations where the target and distractors appeared can be divided into “left or right” or “upper or bottom” areas in Experiment 4, in contrast to Experiment 1, where both target and distractors appeared within the same circle. Such a spatial grouping of the stimuli could increase the distinctiveness of the target and lead to an efficient processing of the target duration. Therefore, we speculate that the amount of attentional interference of the distracting duration with the processing of the target duration may depend on the distinctiveness of the target stimulus.

In addition to attentional accounts of time perception, our results can also be explained by another model. A weighted sum of segment account (Matthews, 2013) postulates that the judged duration of a segmented interval is equal to the sum of the judged duration of the individual segments, with more recent segments having weighted more heavily. This model further predicts that (a) the judged duration of a given interval will increase as the number of segments increases, (b) judged duration will be maximal when the segments are of equal length, and that (c) the overall interval depends only on the size of the segments (Matthews, 2013). In the present study, we observed that the normalized reproduced durations sharply increased when the number of distractors was extremely large (79, in Experiment 4). In light of the first prediction by Matthews (2013), this result could be attributed to the target duration being divided into more segments. The model may also explain our results obtained in Experiments 1 and 2, where the normalized reproduced durations were shorter when the number of distractors increased. This also increased the number of segments presented later in the stimulus window. The larger number of distractors in our experiments may be comparable to the rapid change of the sequences used in Matthews’s model. In sum, in addition to attentional accounts, our results can be interpreted in the framework of the weighted sum of the segments account. However, as our study randomly determined the onsets of the target and distractors, it is hard to know whether/how segmenting the target duration by onsets and offsets of the distractors itself affected the perceived duration of the target. Future studies are needed to address this issue by systematically manipulating the number of segments divided by the distractors, controlling the number of distractors.

How many clocks does the timing system have? Or rather, how much duration information can be processed simultaneously? This question has been frequently discussed in research on time perception. Studies with temporal-adaptation tasks suggest that spatially tuned independent clocks encode the duration information of specific locations (Ayhan et al., 2009; Johnston et al., 2006). More recently, Cheng et al. (2014) demonstrated that the capacity of simultaneous temporal processing was limited to around three or four spatial locations. Such spatially tuned clocks would enable us to selectively time only the relevant duration that is spatially localized, even when the duration is surrounded by irrelevant ones. However, our results (Experiment 2) indicated that the normalized reproduced duration of the target was affected by the physical durations of the surrounding stimuli presented at different locations. This contradicts the assumption that there are multiple clocks, which independently process each of the durations because such clocks would not be influenced by the distractors. Thus, it is unlikely that the target stimulus was assigned a specific spatially tuned clock. Further, recent research suggests that multiple clocks may not be necessary when processing multiple temporal information. Bryce et al. (2015) and Bryce and Bratzke (2016) showed that it is likely that human temporal processing uses a single pacemaker and accumulator, instead of multiple ones, when processing two temporally overlapping intervals. Although the present study does not directly answer the question of how many pacemakers and accumulators are involved in time perception, our results suggest that even if there are multiple pacemakers and accumulators, they are not independent of each other.

While previous studies have shown associations between temporal processing and some areas such as the sensorimotor cortex (Jazayeri & Shadlen, 2015), basal ganglia (Coull et al., 2011), and cerebellum (Lee et al., 2007), the exact neural mechanisms for temporal processing remain unclear (Üstün et al., 2017). Neuroimaging studies have suggested that prefrontal and parietal brain areas are associated with the top-down control of the interference of irrelevant distractors (De Fockert et al., 2004; Gazzaley & Nobre, 2012; Lavie & de Fockert, 2006; Nobre et al., 2000). These brain areas are also involved in the selective processing of relevant duration information and inhibition of irrelevant durations. Because we did not directly measure neural activities and did not find any effects of cortical distances, our results cannot provide evidence regarding the neural correlates related to the interference of the task-irrelevant durations. Future neuroscientific research is required to understand the roles of brain areas related to selective attention in temporal processing.

The present study provides evidence that (a) the processing of the relevant duration became more variable and the perceived duration shortened when the number of surrounding multiple irrelevant durations increased up to around 10, (b) the perceived duration of the relevant stimulus was pulled toward the physical duration of the irrelevant stimuli, (c) an extremely large number of irrelevant stimuli increased the perceived duration of the relevant stimulus, and (d) these effects may not largely depend on early visual processing. Overall, the present study mainly demonstrated that multiple irrelevant duration information affects the temporal processing of relevant duration information and suggests that multiple independent clocks assigned to each of the durations may not exist.
